# Wireless and Battery-Free Sensor for Interstitial Fluid Pressure Monitoring

**DOI:** 10.3390/s24144429

**Published:** 2024-07-09

**Authors:** Chengyang Qian, Fan Ye, Junye Li, Peter Tseng, Michelle Khine

**Affiliations:** 1 Department of Biomedical Engineering, Henry Samueli School of Engineering, University of California Irvine, Irvine, CA 92697, USAjunyel2@uci.edu (J.L.); 2 Department of Electrical Engineering and Computer Science, Henry Samueli School of Engineering, University of California Irvine, Irvine, CA 92697, USAtsengpc@uci.edu (P.T.)

**Keywords:** wireless pressure sensor, LC inductive coupling, heart failure biomarker detection

## Abstract

Congestive heart failure (CHF) is a fatal disease with progressive severity and no cure; the heart’s inability to adequately pump blood leads to fluid accumulation and frequent hospital readmissions after initial treatments. Therefore, it is imperative to continuously monitor CHF patients during its early stages to slow its progression and enable timely medical interventions for optimal treatment. An increase in interstitial fluid pressure (IFP) is indicative of acute CHF exacerbation, making IFP a viable biomarker for predicting upcoming CHF if continuously monitored. In this paper, we present an inductor-capacitor (LC) sensor for subcutaneous wireless and continuous IFP monitoring. The sensor is composed of inexpensive planar copper coils defined by a simple craft cutter, which serves as both the inductor and capacitor. Because of its sensing mechanism, the sensor does not require batteries and can wirelessly transmit pressure information. The sensor has a low-profile form factor for subcutaneous implantation and can communicate with a readout device through 4 layers of skin (12.7 mm thick in total). With a soft silicone rubber as the dielectric material between the copper coils, the sensor demonstrates an average sensitivity as high as –8.03 MHz/mmHg during in vitro simulations.

## 1. Introduction

Almost 7 million people in the United States suffer from heart failure (HF), causing approximately 700,000 deaths every year [1]. HF is a clinical syndrome where the heart fails to provide adequate cardiac output for normal metabolic requirements or to accommodate venous return [2,3]. Notably, approximately a quarter of all HF patients require 30-day hospital readmission after initial diagnosis [4]. From 2015 to 2019, 34.6% of the patients were readmitted to the hospital within 30 days, and according to Medicare, the 1-year HF death rate reached 30% [5,6]. The high readmissions and death rates are due to the progressive severity of HF. If HF is left uncontrolled, it progresses to congestive heart failure (CHF), the end stage of HF where no cure is known. In CHF, fluid accumulates in the lungs, liver, and lower extremities, subsequently resulting in renal complications and other organ dysfunctions [2,3].

The lack of early diagnostic methods profoundly contributes to HF’s high readmission and death rates [7,8]. Tracking the onset during its early stages of decompensation is imperative, as delayed medical intervention advances severity and complexity, leading to increased hospitalization stays, deaths, and total healthcare costs [9,10,11,12]. Despite the importance of early diagnosis, CHF monitoring still relies predominately on subjective patient-reported later-stage symptoms including increased heart rate, shortness of breath (dyspnea), and fluid retention (edema and weight gain) [13]. Therefore, an earlier, pre-symptomatic alert of impending decompensation is still needed.

In clinical settings, diagnostic tests for CHF include blood tests, electrocardiograms, chest X-rays, thoracic impedance measurements, computerized tomography, and magnetic resonance imaging. None of these technologies are amenable to convenient or continuous at-home monitoring [14,15,16,17,18,19]. Portable devices exist for thoracic impedance monitoring, but they require invasive procedures and are primarily designed for patients with other existing implants like pacemakers [20]. Thoracic impedance measurement is usually inaccurate and prone to error [21]. Non-invasive monitoring technologies exist, but they mainly focus on late-stage symptoms like fluid retention/edema [22,23,24,25,26,27,28]. Ideally, CHF-related biomarkers should be continuously monitored, even in outpatients, so that timely intervention can be performed during decompensation onset.

Blood pressure is a potentially viable biomarker for CHF, but it needs to be continuously monitored to offer trends that are useful for CHF prediction [29,30]. The current gold standard for continuous blood pressure monitoring is the arterial line, in which a catheter is placed inside the artery for direct pressure sensing [31]. Inspired by the arterial line, products such as the Chronicle and the HeartPOD leverage catheters inserted into the heart ventricle/atrial for direct measurement of pressure [21,32,33,34]. However, such catheters require power supplies and hardware for pressure information communication that contribute to a large form factor. Unlike those that operate based on an implanted catheter, CardioMEMS is a device with a smaller form factor, and it is implanted into the pulmonary artery (minimally invasive) for pressure sensing [21,35]. However, endothelialization with the vessel wall makes it difficult to remove without damaging the artery [36]. Moreover, all these monitoring devices require skilled professionals for installation and are expensive (>$20,000).

Interstitial fluid pressure (IFP) is a frequently overlooked CHF-related biomarker [1]. During CHF, the heart’s ventricle fails to pump enough blood, resulting in blood accumulation in the veins that eventually causes increased venous pressure [37,38,39]. Increased venous pressure consequently leads to increased capillary hydrostatic pressure, causing the extravasation of electrolytes and fluid from the capillary into the interstitium [39]. When the extravasation outworks the lymphatic system’s capacity to return fluid to the vascular space, IFP increases [37]. Importantly, IFP increases by approximately 7 mmHg in CHF patients before any observable edema can be detected [40,41,42]. If the change in IPF exceeds 7 mmHg, observable edema is detected in the lungs or lower limbs [37,40]. While sensors exist for continuous peripheral edema monitoring via tracking swelling using various sensing modalities and a positive relationship between edema and IFP exists, observable edema implies that IFP has already far exceeded the normal value [22,23,24,25,26,27,28]. Importantly, by the time a patient exhibits edema, the heart’s pumping capability has reduced to one-third of its original value [40,41]. Therefore, direct IFP monitoring offers an earlier and better indicator of decompensation than monitoring for edema.

Existing methods for direct IFP measurement typically involve inserting a catheter with a built-in transducer into the area of interest [42,43,44,45,46,47]. However, these methods require catheter insertion into the body while acquiring IFP information; long-time catheter insertion might cause complications such as infection [48,49]. Therefore, current methods are not suitable for continuous IFP monitoring for CHF prediction.

**Figure 1:**
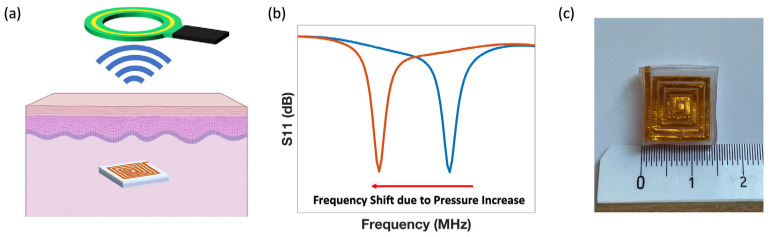
(**a**) Schematic of sensing mechanism. The subcutaneously implanted sensor communicates with external readout coil via inductive coupling. (**b**) An example of resonant frequency change due to pressure change. (**c**) A picture of the sensor.

The research progress in the field of wearable electronics has the potential to revolutionize the current methods of relevant biomarker monitoring and healthcare delivery [50]. The integration of point-of-care sensors and diagnostics on soft and flexible substrates minimizes the stiffness mismatch with body tissue and leads to more comfortable wearability of those devices for long-term use, regardless of whether those devices are implanted or non-invasive [51,52,53]. The use of serpentine metal trace, surface microstructure, nanowires/tubes, liquid metals, etc., on soft and flexible substrates enables the formation of sensors/conductive paths that can accommodate certain strains and achieve conformal contact with interfacing tissue to minimize motion artifacts and obtain high-resolution signal [54,55,56,57,58,59,60,61,62,63,64,65]. It has already been demonstrated that wearable pressure and strain sensors could provide equivalently reliable blood pressure and respiratory rate monitoring compared with their respective gold standards [56,60,63,65,66]. Miniaturization of wearable sensors will further amplify their advantages since their small size will reach conformal contact with tissue more easily, and their soft substrates can minimize the immune response if implanted [67]. In the context of this paper, miniaturized wearable pressure sensors are most suitable for continuous IFP monitoring as their small sizes also allow minimally invasive implantation, enabling continuous IFP monitoring directly. Implanting them subcutaneously is much easier compared to catheter-based devices. However, not all sensing modalities are suitable for this purpose. Piezoresistive and optical pressure sensors usually require batteries to power the system [45,68,69,70,71]. Battery life and size/weight make implantation impractical. Piezoelectric pressure sensors do not require batteries, but they are best suited for measuring dynamic pressure input, not a gradually increasing pressure like IFP [72]. Capacitive pressure sensors have gained great popularity for their simple device design and ease of integration with inductors to form LC pressure sensors that do not require batteries as implants and can deliver pressure information wirelessly [23,73,74,75,76,77,78,79,80,81,82,83,84,85,86,87,88,89,90,91,92,93]. The resonance frequency of an LC circuit is defined as:

(1) $$f=\frac{1}{2\pi \sqrt{LC}}$$

where L is the sensor inductance, C is the sensor capacitance and f is the sensor resonant frequency. When pressure is applied due to an increase in interstitial fluid pressure, both the capacitance and the inductance of the sensor increase, leading to a decrease in the resonant frequency of the implanted sensor [87]. This decrease in resonant frequency can be detected wirelessly by an external readout coil (Figure 1a), allowing for the wireless detection of pressure changes (Figure 1b).

We introduce a minimally invasive LC-type sensor for IFP monitoring. An asymmetrical design is adopted for the top and bottom coil (top coil is clockwise and bottom coil is counterclockwise) since this design has been shown to result in a larger change in resonant frequency (Figure 1c) [87,88]. The sensor is composed of copper coils. Those coils are made from the copper sheet (CTF-3, 11.85 × 7.99 inches, 25 μm thick, Vootape, Amazon, Seattle, WA, USA) and they are defined by a craft cutter (Silhouette Cameo 4 system, Silhouette America, Lindon, UT, USA) on double-sided Kapton tape (Double-Sided Polyimide High-Temperature Tapes, Kaptononline, Torrance, CA, USA) stacked on PDMS sheet (Silicone Sheet, 0.25 mm thick, AAA-ACME Rubber Company, Tempe, AZ, USA). The planar coils are folded across the middle line to form an LC-type sensor, and a 3 mm thick Ecoflex layer (Ecoflex 00-30, Smooth-on, Macungie, PA, USA) is used as their separation layer. The sensor’s simple design makes it suitable for inexpensive batch production. Furthermore, complicated fabrication techniques and materials are not required. Since soft Ecoflex is used as the dielectric material, the sensor has an average sensitivity of −3.11 MHz/mmHg. It has an acceptable form factor and can communicate with a readout coil through 4 layers of pork skin (12.7 mm thick in total). Pork skin is frequently used to simulate wearable device performance in vivo due to its anatomical and physiological similarities with human skin [87,94,95,96,97]. To simulate IFP increase in vivo, sensors were sandwiched between two pieces of fresh pork (10 mm each) and demonstrated an average sensitivity of −8.03 MHz/mmHg.

## 2. Materials and Methods

### 2.1. Materials

PDMS sheet (Silicone Sheet, 0.01 inch thick) was purchased from AAA-ACME Rubber Company (Tempe, AZ, USA). PDMS and curing agent (Sylgard 184) was bought from Dow (Midland, MI, USA). The acrylic sheet (Polycast 84, 6 mm thick) was bought from Spartech (Stamford, CT, USA). Double-sided Kapton tape (Double-Sided Polyimide High-Temperature Tapes, 4 ml thick) was purchased from Kaptononline (Torrance, CA, USA). Ecoflex (Ecoflex 00-30) was bought from Smooth-on (Macungie, PA, USA). Sponge (Sponge Model 40102) was purchased from Carrand Companies (Carson, CA, USA). The copper sheet (CTF-3, 11.85 × 7.99 inches, 25 μm thick) was bought from a retailer called Vootape on Amazon (Seattle, WA, USA). Desiccants (Drierite Drying Desiccants) were purchased from W.A. Hammond Drierite Co. (Xenia, OH, USA). Fresh pork skins (average thicknesses were 3.2 mm for the separation distance test and 10 mm for the sensitivity characterization test, respectively) were bought from the local supermarket (Irvine, CA, USA).

### 2.2. Sensor Fabrication

**Figure 2:**
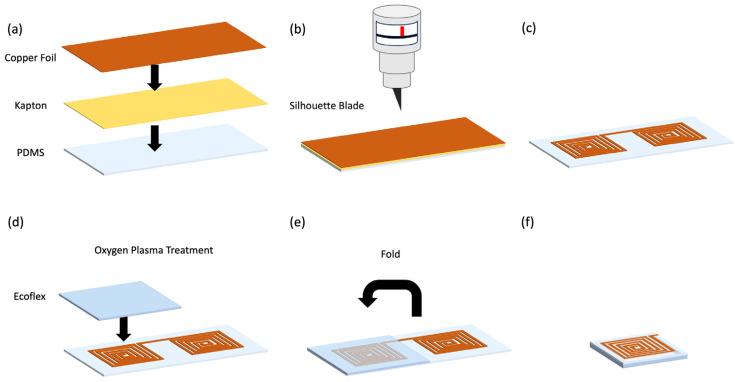
Sensor fabrication flow. (**a**) Laminating copper sheet on top of double-sided Kapton tape and PDMS film. (**b**) Cutting the copper sheet into coils using the Silhouette Cameo 4 System. (**c**) The layout of the planar coil. (**d**) Assembly of Ecoflex dielectric after oxygen plasma treatment. (**e**) Folding the sensor across the middle line. (**f**) The completed sensor.

The sensor fabrication flow is demonstrated in Figure 2. The copper sheet was laminated on top of double-sided Kapton tape, and then the double-sided Kapton tape was laminated on top of PDMS film (Figure 1a). Copper coils with the desired shape were defined using the Silhouette Cameo 4 system (Figure 2b,c). The gap between the turns was kept at 0.25 mm for all sensor form factors since this was the minimal size achievable by the craft cutter. The speed and force were set to 4 and 12, respectively. The depth of the cut was determined by the length of the adjustable blade, and it was set to 2 (~0.2 mm protrusion). Combined with selected speed and force, this blade length could cut through the copper foil and Kapton layer without damaging the underneath PDMS substrate. The entire substrate was treated with oxygen plasma at 200 mTorr for 60 s and folded across the middle line so that the Ecoflex could bond with the PDMS substrate (Figure 2d–f).

Sponge, Ecoflex (A:B = 1:1), and PDMS (monomer: curing agent = 10:1) dielectric were made for sensitivity characterization and comparison. All of them were 3 mm thick.

### 2.3. Instrumentation and Characterization

A force gauge (Force Gauge Series 5, Mark-10 Corporation, Copiague, NY, USA) was connected to a test stand (ESM303, Mark-10 Corporation, Copiague, NY, USA) so that known normal forces were applied and relevant pressure could be calculated. An LCR meter (E4980AL Precision LCR Meter, Keysight, Santa Rosa, CA, USA) was used to obtain capacitance change during the cyclic fatigue test. VNA (E5063A, Keysight, Santa Rosa, CA, USA) was used to capture the resonant frequency shift when different pressures were applied. A plasma cleaning system (PE-50, Plasma Etch, Carson, NV, USA) was used to provide oxygen plasma. A one-loop coil (15 mm diameter near field probe, Fafeicy, China) was used as the readout coil. A craft cutter (Silhouette Cameo 4 system, Silhouette America, Lindon, UT, USA) was used to cut the sheet into the desired coil shapes and it used a blade with adjustable length (Silhouette AutoBlade, Lindon, UT, USA). Humidity around the sensor was obtained from a hygrometer (Mini Temperature and Humidity Meter, Tasogen, China). An iPhone 12 Pro (Apple, Cupertino, CA, USA) was used as a source of electromagnetic noise so that sensors’ resistance to electromagnetic interference could be tested. The acrylic sheet was cut using a laser (Versa Laser, 1060 nm, Scottsdale, AZ, USA) to define different arcs with different curvatures. The sensor adhered onto the arcs using double-sided tapes so that it could be bent by different angles and the corresponding frequency shifts could be observed.

All data analysis and graphing were performed in MATLAB (R2023a, MathWorks, Portola Valley, CA, USA). Electromagnetic simulations were carried out with CST Microwave Studio Suite (2022 Version, Dassault Systems, Velizy-Villacoublay, France).

## 3. Results and Discussion

### 3.1. Sensor Form Factor Determination

**Figure 3:**
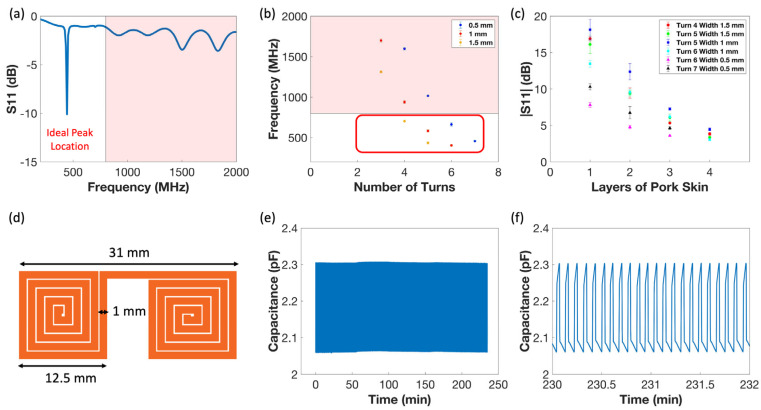
(**a**) The background of the readout coil and one representative resonant peak is below 800 MHz. The shaded area indicates the frequency range where obvious background frequency peaks are present. (**b**) The resonant frequencies of different sensor designs. The shaded area indicates the frequency range where obvious background frequency peaks are present. The red box includes sensors whose resonant frequencies are below 800 MHz. Those that are not included in the red box have resonant frequency over 800 MHz, overlapping with the background peaks. (**c**) The observed resonant frequency peak heights through various layers of pork skin. All the error bars in (**b**,**c**) are the calculated standard errors. (**d**) The chosen sensor design: side length 12.5 mm and coil with 1 mm. (**e**) Capacitance change of the sensor during 2500 cycles of loading and unloading. (**f**) Zoomed-in waveform of the sensor during cyclic test.

Different sensor designs were fabricated following the procedure explained in Section 2.2 and tested to determine the sensor with the smallest form factor capable of communicating through the tissue. Sensors with different numbers of turns and coil widths were made (n = 3 for each sensor form factor), and a one-loop readout coil was used to find their resonant frequencies. Due to the self-resonance of the readout coil, it contributes to peaks in the frequency spectra background, and these peaks could overlap with the sensor’s resonant frequency peak. Those overlaps make the detection of sensor operation more difficult [86,98,99,100]. The background of the readout coil has obvious peaks beyond 800 MHz, as shown in the shaded area in Figure 3a. The sensor’s preferred resonant frequency was below 800 MHz to avoid overlap with peaks in the background. Additionally, implanted sensors will have poor inductive coupling with readout coil and low signal penetration depth in tissue if their resonant frequencies are within the high MHz to GHz region [101]. Consequently, those sensors whose resonant frequencies were above 800 MHz were screened out (Figure 3b).

The remaining sensors were further tested to determine if they could communicate through different layers of pork skins (3–5 mm each layer). S11 peak heights were collected at each separation distance for each form factor. Those sensors with 0.5 mm coil width could not generate detectable peak possibly due to their high coil resistances (Figure 3c). High resistance meant more energy was dissipated in the sensor than reflected to the readout coil. Even less energy was transmitted when the separation distance was increased and hence, the S11 peak eventually became undetectable for those sensors. Among the rest of the sensors, the sensor with five turns and a coil width of 1 mm was identified as the smallest sensor capable of communicating with the readout coil through four layers of pork skin. The total thickness of the pork skin was roughly 12.7 mm. Edema is usually first observed in lower limbs, and the average subcutaneous tissue thicknesses in the legs are 10.79 mm in males and 12.44 mm in females, respectively [102]. Therefore, the sensor can communicate with the readout coil even when implanted subcutaneously in lower limbs. The detailed form factor of this sensor design is demonstrated in Figure 3d.

A cyclic fatigue test was also performed to demonstrate the durability of the sensor. The planar coil was cut in the middle so that coils served as a capacitor and its capacitance could be continuously captured during the fatigue test using an LCR meter. Approximately 50 kPa of compressive pressure was applied upon the sensor for 2500 times of loading and unloading (Figure 3e,f). For each cycle, the changes in slope were due to the incompressibility of the Ecoflex when force was applied and the slow relaxation of material when force was released, respectively. No significant amplitude or baseline changes were observed, suggesting that the sensor can potentially withstand repeated pressing and still provide a stable signal reading after implantation.

### 3.2. Sensor Sensitivity Characterization

**Figure 4:**
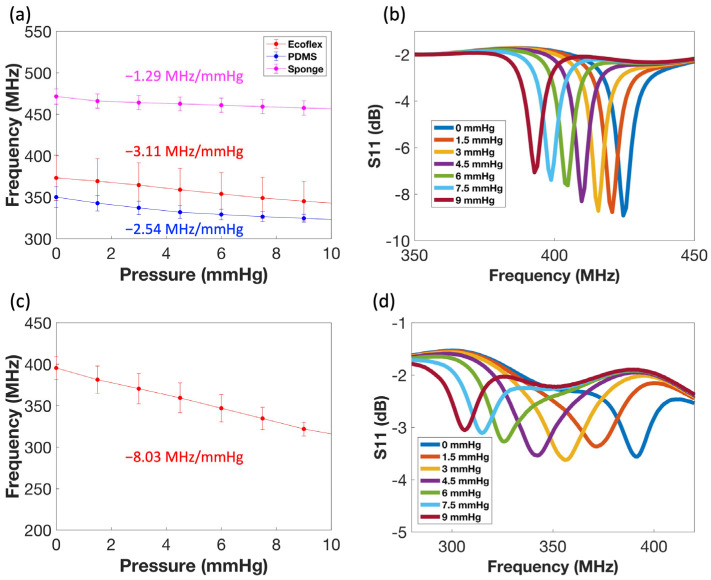
(**a**) Averaged sensitivity curves at low-pressure range. (**b**) A representative sensor resonant frequency shifts under various applied pressures at a low-pressure range. (**c**) Averaged sensitivity curve at low-pressure range when sandwiched between 2 pieces of pork skins. (**d**) A representative sensor resonant frequency shifts under various applied pressures at a low-pressure range when sandwiched between 2 pieces of pork skins. All the error bars are the calculated standard errors.

The LC sensors (n = 3 for each dielectric material) were fabricated using Ecoflex, PDMS, and sponge as dielectric materials for sensitivity comparison. The purpose of the sponge was to prevent the coils from contacting each other. External pressure loads were applied to the sensors, and their corresponding resonant frequency changes were captured. The sensors that used Ecoflex as dielectric material showed an averaged sensitivity as high as −3.11 MHz/mmHg in the low-pressure range and the sensitivity decreased to −0.104 MHz/mmHg as Ecoflex gradually became incompressible (Figure 4a and Supplementary Materials Figure S1a). These sensors demonstrated higher sensitivity than those that used sponge and PDMS as dielectric materials in the low-frequency range due to Ecoflex’s higher dielectric constant than sponge and softer mechanical properties than PDMS, respectively.

A representative sensor’s resonant frequency shift under various applied pressures is shown in Figure 4b, demonstrating the sensor’s high sensitivity at the low-pressure range. The high sensitivity of our sensor is probably due to the asymmetrical coil layout and the applied pressure brought the two copper coils closer to each other, causing the effective coupling capacitance to increase and shift the resonant peak more significantly. Similar sensor designs are presented in other works, demonstrating their sensors’ high sensitivity as well [86,87,88].

Next, the sensors were sandwiched between two pieces of pork skin to demonstrate their capability of capturing pressure change as subcutaneous implants. The sensors were placed between two layers of pork skin, each roughly 10 mm thick. The same pressures were applied again, and the corresponding resonant frequency changes were also captured. The sensors’ averaged sensitivity increased to −8.03 MHz/mmHg, possibly due to the presence of water in the pork skin, which increased the dielectric constant around the sensor that led to higher capacitance change (Figure 4d and Supplementary Materials Figure S1b).

An example of a sensor’s resonant frequency shift under various applied pressures is shown in Figure 4d. Shifts were still observed, despite the peaks becoming shorter and slightly distorted. The depth and sharpness of the S11 peaks were defined by Q-factor, a quantity defined by the following equation:

$$Q=\frac{1}{R}\sqrt{\frac{L}{C}}$$

where R, L, and C are the resistance, inductance, and capacitance of the sensor, respectively. In this case, shorter and more distorted peaks indicated a low Q-factor, due to the presence of water. Nevertheless, compared with other published sensor designs, our sensor design had higher sensitivity at the low-pressure range [75,79,80,81,82,83,84,85,86,87,88,103,104].

The sensor’s resonant frequency change in response to humidity change was also studied. A beaker filled with water was placed in an enclosed box so that the humidity in the box could be ramped up to 80% due to the evaporation of water and then, the beaker was removed. The sensor, desiccant and hygrometer were placed in an enclosed box. Due to the presence of desiccant, the humidity inside of the box dropped from 80% to 22%, and the corresponding resonant frequency at each humidity was recorded. The sensor’s resonant frequency did not change because of humidity change possibly due to the hydrophobic surface of PDMS/Ecoflex repelled water (Supplementary Materials Figure S2). The result indicated that the sensor could provide relatively stable resonant frequency despite some humidity changes around its local environment. Nevertheless, the sensor should experience an environment already saturated with water once implanted in the interstitial space. While inside the body, the sensor should experience liquid water (100% humidity) in its local environment. Even when IFP increases, having more water in the interstitial space still ensures the sensor is surrounded by water (100% humidity). As such, the resonant frequency should not shift due to humidity change once inside the body since humidity around the sensor should not change.

The sensor’s ability to resist electromagnetic interferences was tested by placing an iPhone 1 cm above the sensor and the readout coil. The resonant frequency of the sensor was recorded initially without the presence of the phone and then with the phone placed above the sensor. The phone was set to different modes (resting, playing YouTube, and with a phone call) so that different levels of interference could be imposed onto the system. Resonant frequency at each mode was recorded; no shift in resonant frequency was not observed (Supplementary Materials Figure S3a). The result indicated that the sensor’s resonant frequency shift will not be confounded by everyday levels of electromagnetic interference. The sensor’s tolerance to bending motion was also tested by adhering the same sensor onto acrylic arcs with different curvatures using double-sided tape. Different arcs provided different degrees of bending so that frequency shifts could be observed at each bending angle. The sensor experienced left shifts; bending motions may also induce dielectric material compressions or elongation, resulting in larger capacitances (Supplementary Materials Figure S3b). Although these unwanted shifts could be a confounding factor, the shift per degree was low (−1.25 MHz/degree) compared with frequency shift due to normal pressure and enormous force had to be imposed to bend the sensor due to its small size. To minimize the effect of bending, the sensor can be implanted in places where muscle flexion and movement are minimal.

Simulations were performed to verify an asymmetrical coil layout contributes to higher sensitivity. In the simulations, field distributions were determined through the finite-difference time-domain (FDTD) method, employing discrete port excitation. This process utilized a frequency domain solver and tetrahedral mesh, along with open boundary conditions in every direction. Due to the enormous complexity of the model, the application of pressure on the sensor was simulated as a small stepwise decrease in the thickness of the sensor’s interlayer, resulting in higher equivalent inductance and lowered resonant frequency, as previously published [105] (Supplementary Materials Figure S4a,b). Compared with the symmetrical coil layout, the asymmetrical design had a higher frequency shift for each stepwise decrease in dielectric thickness according to the simulation results. Qualitatively speaking, the asymmetrical design had higher sensitivity since the top and the bottom coil formed a quasi-closed coil system that could more effectively leverage the increase in coupling capacitance as the coils were brought closer [86,88]. From an implantation perspective, asymmetrical design was also more desirable since its lower resonant frequency would experience less signal attenuation across tissue [88,101].

A similar simulation setup was adopted for the sensor sandwiched between pork skins so that the sensors’ enhanced sensitivity due to the presence of water in pork skins could be verified. Due to water’s high permittivity, the simulated sensor demonstrated higher sensitivity (Supplementary Materials Figure S5a,b). Both simulation results corroborated well with their corresponding experimental results.

## 4. Conclusions

In summary, we designed an LC-type sensor that can sense pressure changes wirelessly without the use of a battery. The low-profile, minimally invasive sensor has high sensitivity and can be fabricated using simple fabrication techniques. Through various tests, the sensor demonstrated it can communicate through several layers of skin, and therefore, is suitable as a subcutaneous implant for capturing changes in interstitial fluid pressure as an indicator of early congestive heart decompensation.

## Data Availability

All the data created in this study are available upon request.

## Supplementary Materials

The following supporting information can be downloaded at: https://www.mdpi.com/article/10.3390/s24144429/s1, Figure S1: (**a**) Averaged sensor sensitivity curves at low-pressure range. PDMS, Ecoflex and sponge were used as dielectric materials, respectively. (**b**) Averaged sensor sensitivity curve at low-pressure range when sandwiched between 2 pieces of pork skins. These sensors used Ecoflex as dielectric material. Figure S2: The Sensor’s humidity test setup and its resonant frequencies at different humidity levels. Figure S3: (**a**) The resonant frequencies under the influence of electromagnetic noises. The source of noise was an iPhone. The resonant frequencies were recorded both in the absence and presence of the iPhone. The iPhone was also playing YouTube and making phone call so that different electromagnetic noise levels were imposed. (**b**) The sensor’s resonant frequencies different bending angles. Figure S4: (**a**) Simulated resonant frequency change at each dielectric material thickness for sensor with asymmetrical coil layout. (**b**) Simulated resonant frequency change at each dielectric material thickness for sensor with symmetrical coil layout. Figure S5: (**a**) Simulated resonant frequency change at each dielectric material thickness when sandwiched between two pieces of PMMA (**b**) Simulated resonant frequency change at each dielectric material thickness when sandwiched between two pieces of pork skin.
