# Supplementary material for: Wireless and Battery-Free Sensor for Interstitial Fluid Pressure Monitoring
Source: Sensors (Basel). 2024 Jul 9;24(14):4429. doi: 10.3390/s24144429 (PMC11280719; doi:10.3390/s24144429)
Supplement: Supplementary file 1 [file sensors-24-04429-s001.zip › sensors-3062335-supplementary.pptx]

## Slide 1
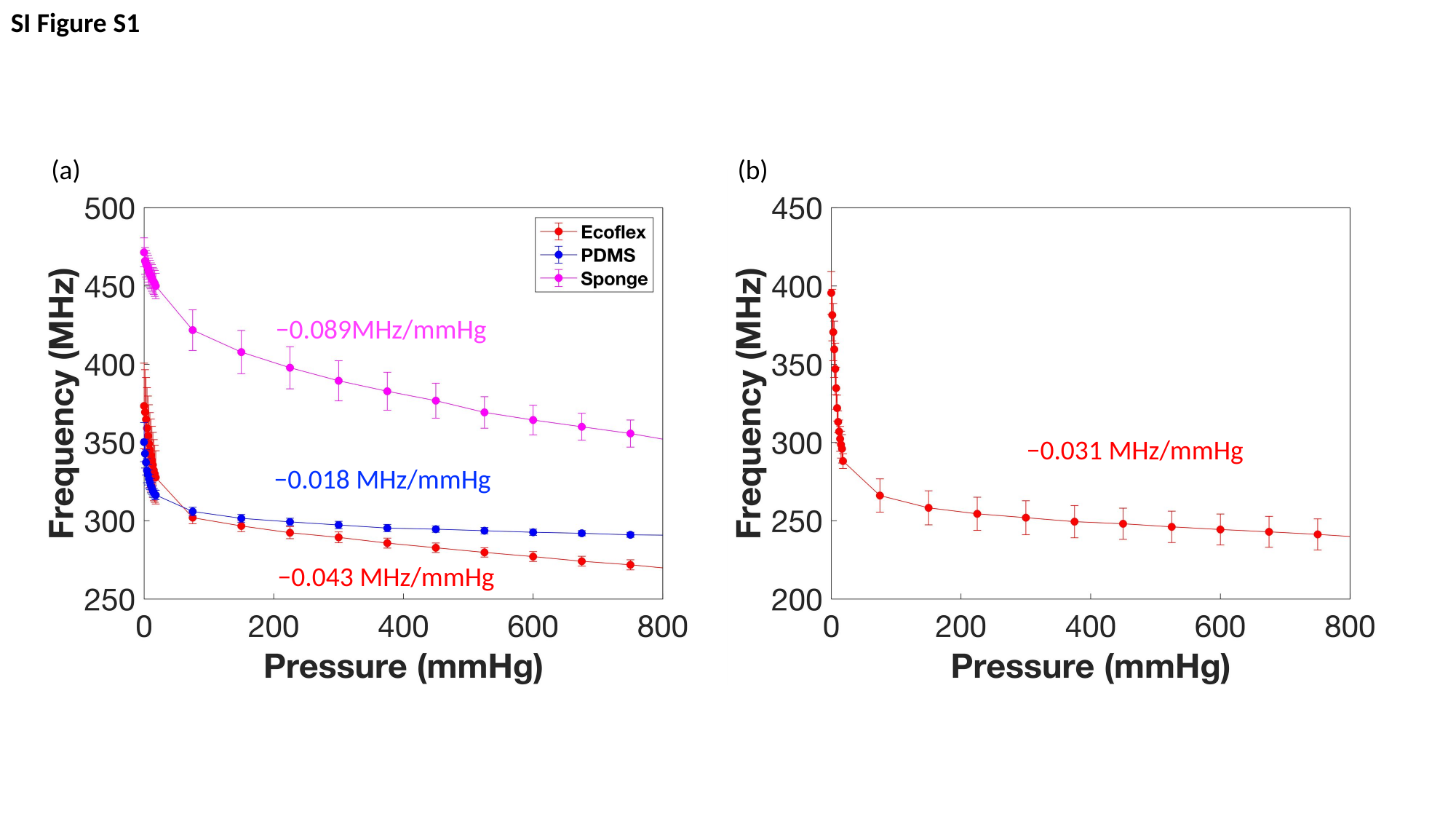

SI Figure S1
(a)
(b)
−0.089MHz/mmHg
−0.031 MHz/mmHg
 −0.018 MHz/mmHg
 −0.043 MHz/mmHg

## Slide 2
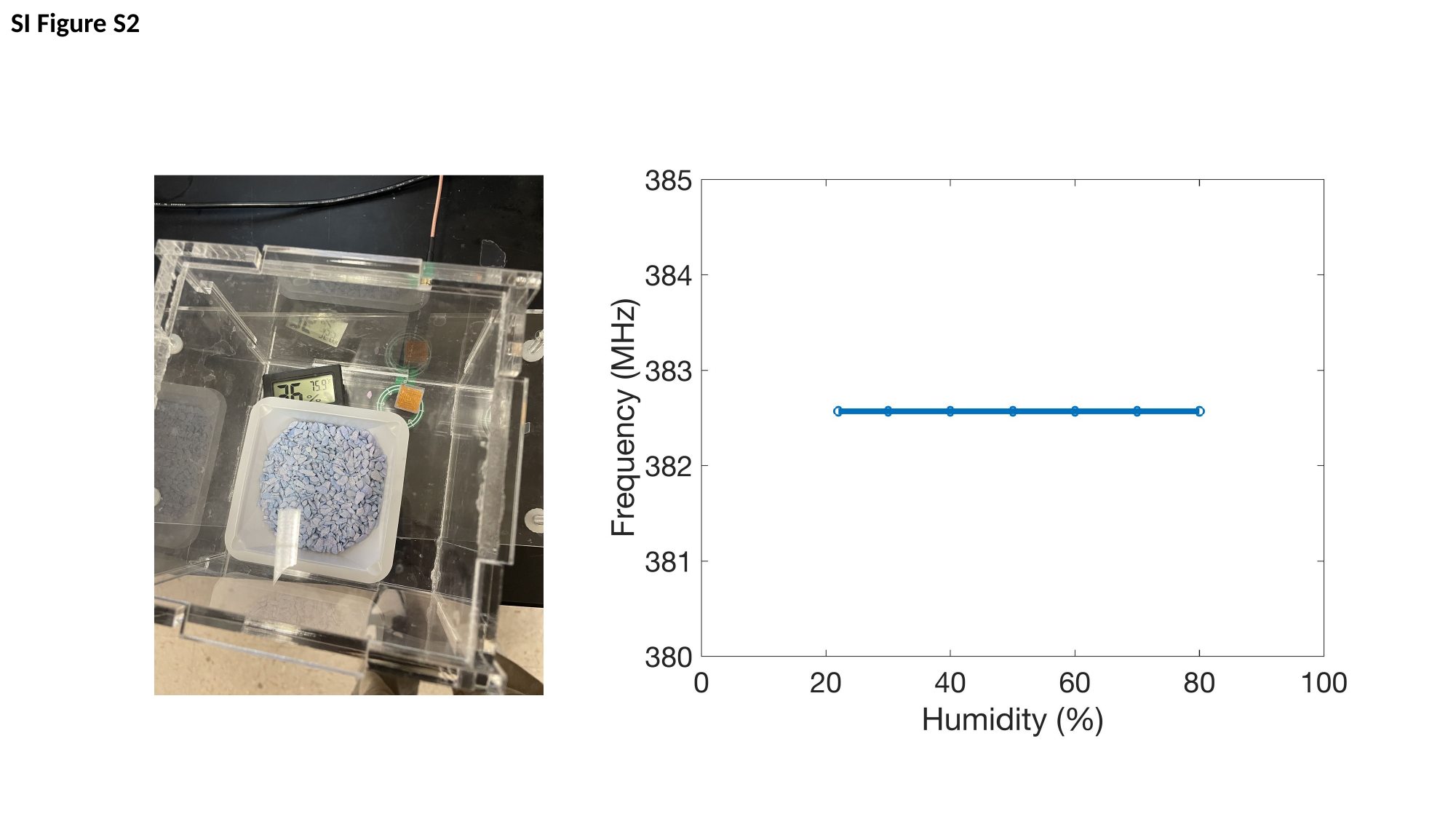

SI Figure S2

## Slide 3
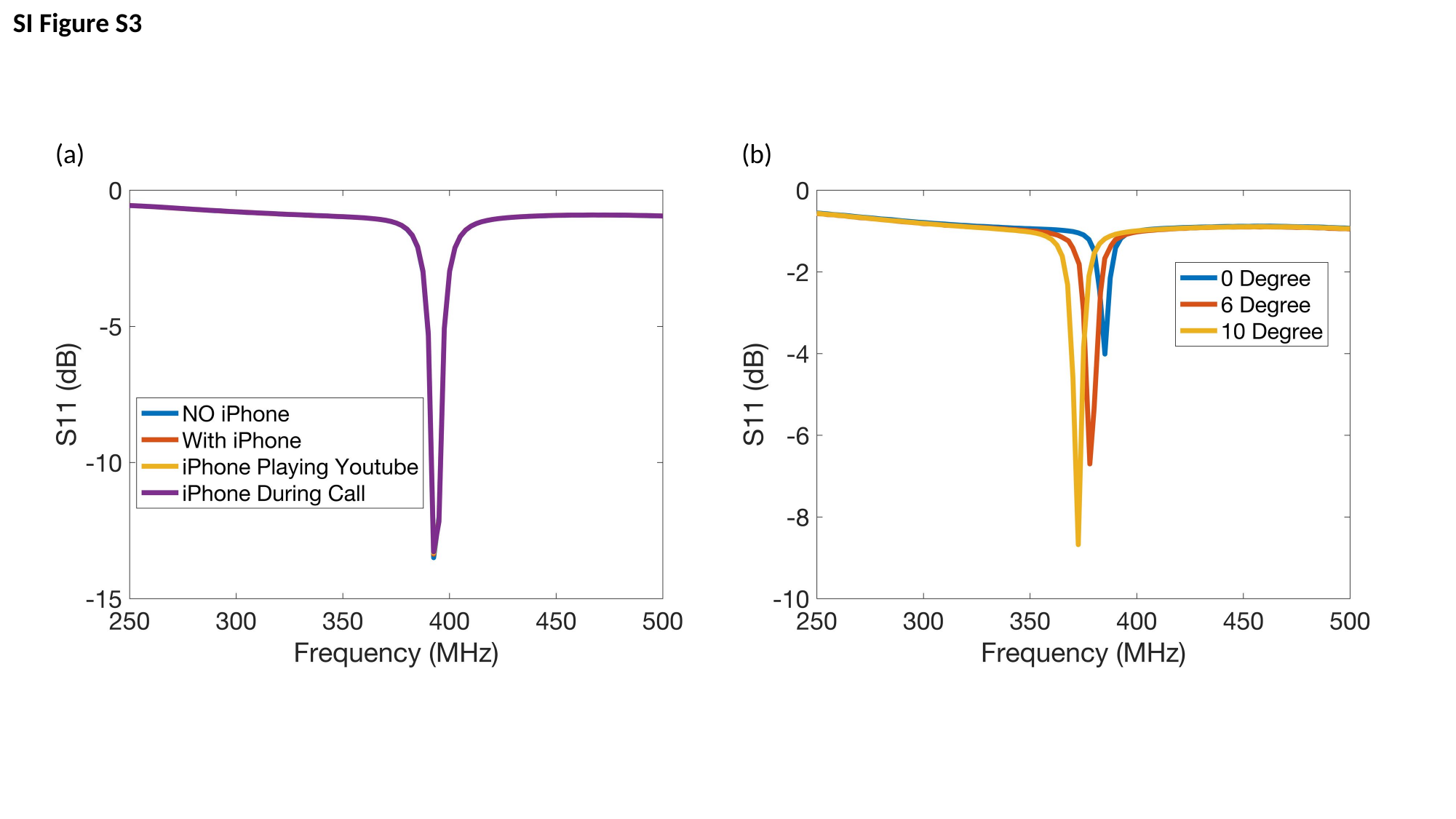

SI Figure S3
(a)
(b)

## Slide 4
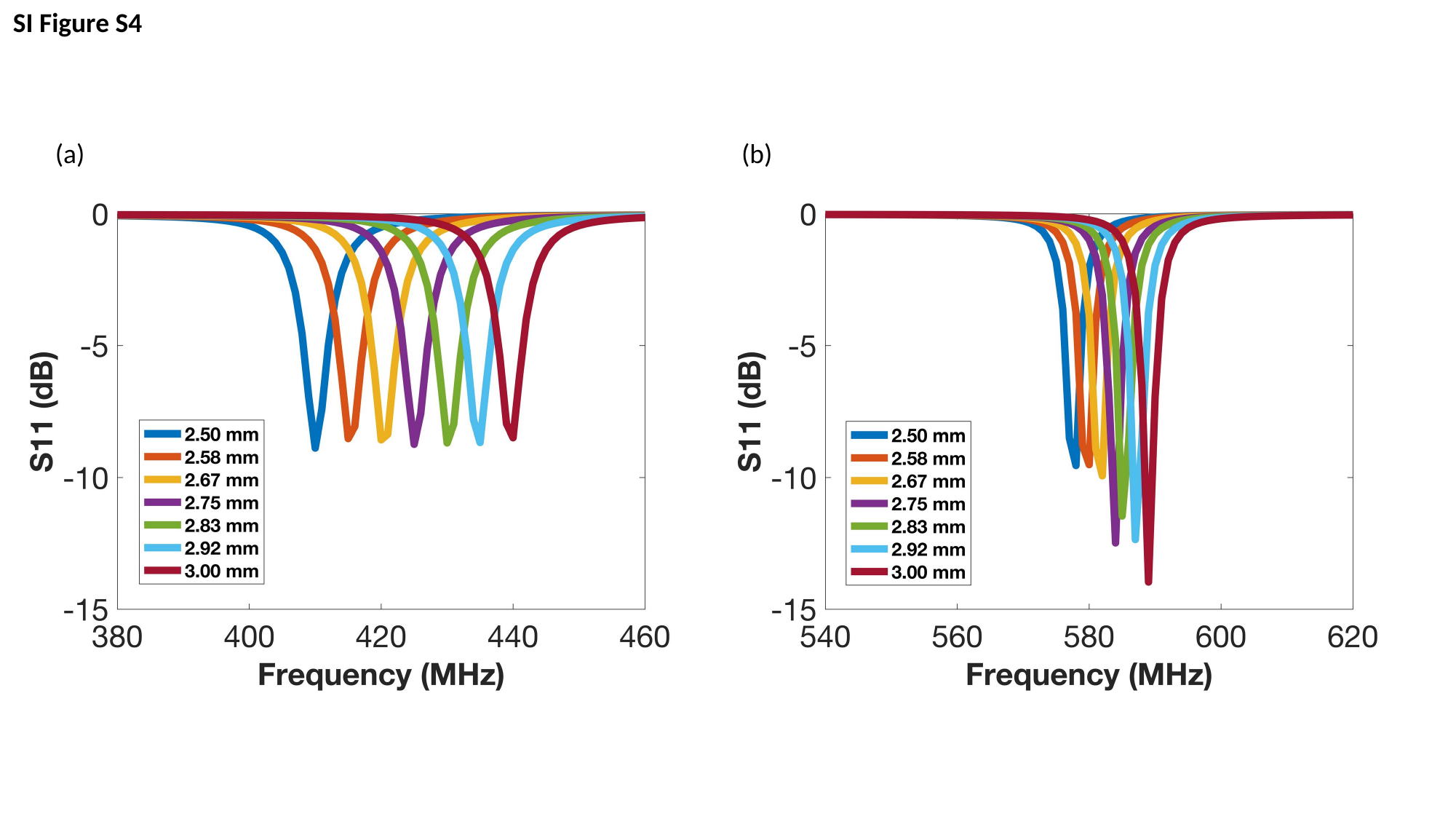

SI Figure S4
(a)
(b)

## Slide 5
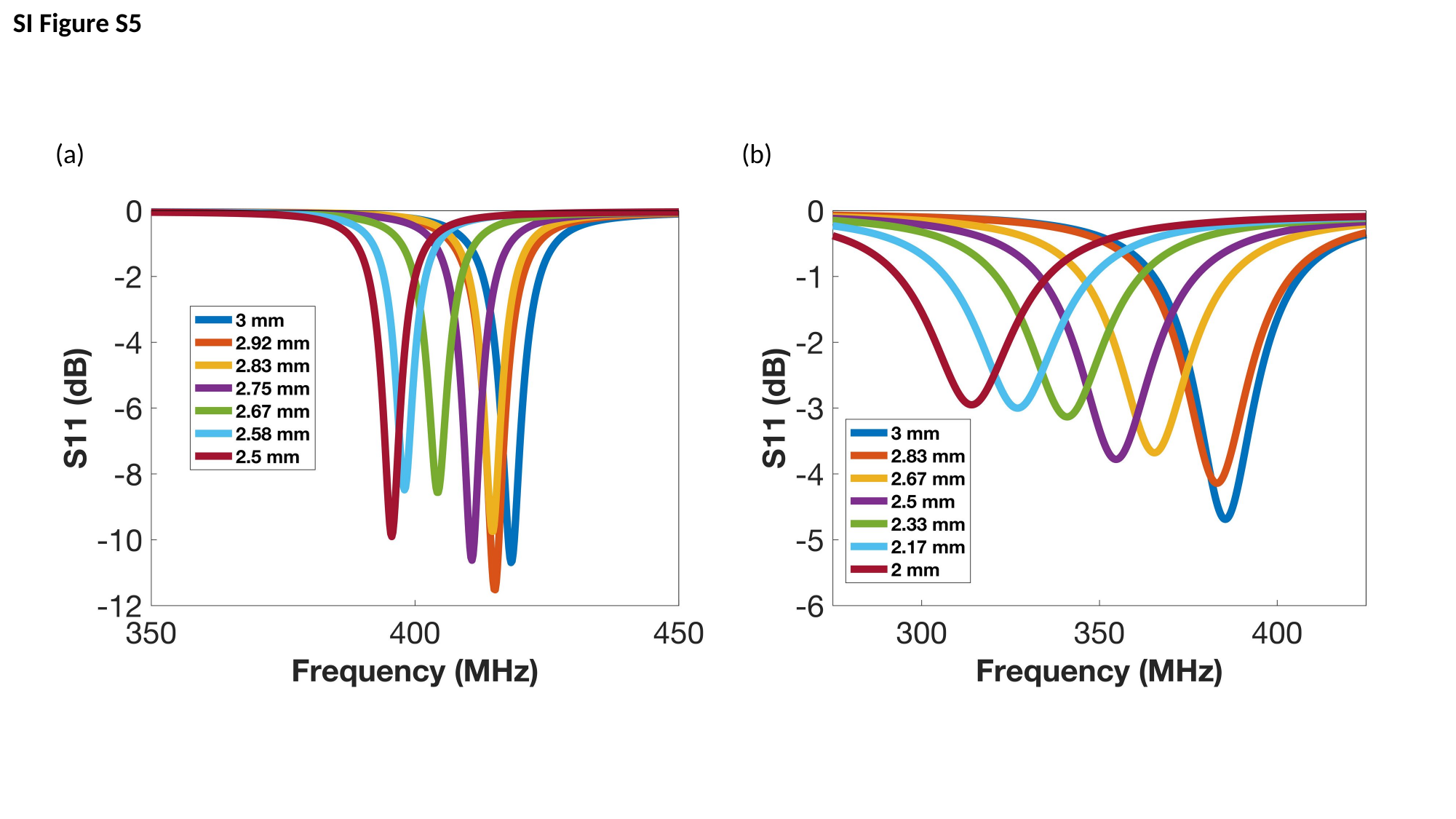

SI Figure S5
(a)
(b)
